# Roe Protein Hydrolysates of Giant Grouper (*Epinephelus lanceolatus*) Inhibit Cell Proliferation of Oral Cancer Cells Involving Apoptosis and Oxidative Stress

**DOI:** 10.1155/2016/8305073

**Published:** 2016-04-18

**Authors:** Jing-Iong Yang, Jen-Yang Tang, Ya-Sin Liu, Hui-Ru Wang, Sheng-Yang Lee, Ching-Yu Yen, Hsueh-Wei Chang

**Affiliations:** ^1^Department of Seafood Science, National Kaohsiung Marine University, Kaohsiung 81157, Taiwan; ^2^Department of Radiation Oncology, Faculty of Medicine, College of Medicine, Kaohsiung Medical University, Kaohsiung 80708, Taiwan; ^3^Department of Radiation Oncology, Kaohsiung Medical University Hospital, Kaohsiung 80708, Taiwan; ^4^Department of Radiation Oncology, Kaohsiung Municipal Ta-Tung Hospital, Kaohsiung 80708, Taiwan; ^5^Department of Biomedical Science and Environmental Biology, Kaohsiung Medical University, Kaohsiung 80708, Taiwan; ^6^School of Dentistry, Taipei Medical University, Taipei 11031, Taiwan; ^7^Division of Orthodontics, Wan-Fang Medical Center, Taipei Medical University, Taipei 11648, Taiwan; ^8^Department of Oral and Maxillofacial Surgery, Chi-Mei Medical Center, Tainan 71004, Taiwan; ^9^Institute of Medical Science and Technology, National Sun Yat-sen University, Kaohsiung 80424, Taiwan; ^10^Center for Research Resources and Development of Kaohsiung Medical University, Kaohsiung 80708, Taiwan; ^11^Cancer Center, Kaohsiung Medical University Hospital, Kaohsiung Medical University, Kaohsiung 80708, Taiwan

## Abstract

Roe protein hydrolysates were reported to have antioxidant property but the anticancer effects were less addressed, especially for oral cancer. In this study, we firstly used the ultrafiltrated roe hydrolysates (URH) derived from giant grouper (*Epinephelus lanceolatus*) to evaluate the impact of URH on proliferation against oral cancer cells. We found that URH dose-responsively reduced cell viability of two oral cancer cells (Ca9-22 and CAL 27) in terms of ATP assay. Using flow cytometry, URH-induced apoptosis of Ca9-22 cells was validated by morphological features of apoptosis, sub-G1 accumulation, and annexin V staining in dose-responsive manners. URH also induced oxidative stress in Ca9-22 cells in terms of reactive oxygen species (ROS)/superoxide generations and mitochondrial depolarization. Taken together, these data suggest that URH is a potential natural product for antioral cancer therapy.

## 1. Introduction

Oral cancer is the sixth most common cancer in the world [1, 2]. Although some oral tumor markers had been identified for detection [3], the oral cancer risk was unable to be reduced due to its nontherapeutic function. Oral carcinogenesis is a complex and long-term multifocal process [4]. Therefore, the drugs with chemoprevention are still needed for antioral cancer therapy.

Fish protein hydrolysates from cobia (*Rachycentron canadum*) [5, 6], tilapia (*Oreochromis* spp.) [7], grass carp (*Ctenopharyngodon idellus*) [8], fresh water carp (*Catla catla*) [9], and other species [10] exhibited the antioxidant property. Furthermore, the roe protein hydrolysates from defatted skipjack (*Katsuwonous pelamis*) [11],* Channa striatus*,* Labeo rohita* [12],* Cyprinus carpio*, and* Epinephelus tauvina* [13] had been found to possess the antioxidant property. These antioxidant properties may have the health promoting effects, such as anti-inflammation and antibacterial [14, 15]. However, the anticancer effect of roe protein hydrolysates-derived antioxidants may have different properties and is less addressed.

Drugs and natural products with the antioxidant effects also reported to inhibit cancer cell proliferation. For example, the grape seed extracts [16], red algal methanol extract [17, 18], and red algal ethanol extract [19] had been reported to be antiproliferative to oral cancer cells. Accordingly, the possible antiproliferative effect of roe protein hydrolysates is warranted for further investigation.

Recently, the protein hydrolysates of fish [20, 21], marine [22, 23], and plant [24] sources have been applied to cancer therapy study. For example, fish protein hydrolysates were found to inhibit proliferation of human breast cancer (MCF-7/6 and MDA-MB-231) cells [20]. Fractions from loach protein hydrolysates prepared by papain digestion have been reported to have the antioxidant and antiproliferative activities against colon (Caco-2) cancer cells [21]. Antioxidant and antiproliferative activities also have been reported in protein hydrolysate of blood clam (*Tegillarca granosa*) muscle against prostate, lung, and cervical cancer cells [22], bioactive peptides from enzymatic hydrolysate of oyster (*Saccostrea cucullata*) against colon cancer cells [23], and bioactive peptides from chickpea (*Cicer arietinum* L.) against breast cancer cells [24]. However, the performance of these protein hydrolysates in oral cancer cells remains unclear.

Giant grouper is the largest specie of groupers in Taiwan. The diameter of a fresh roe is from 2 to 3 mm. Due to its fast growth and high price, giant grouper currently is regarded as a favorite species for marine culture in Taiwan [25]. However, during the massive seed production, a large number of roes have been collected because they failed to hatch. To make the use of the protein byproduct, the enzymatic hydrolysis can be implemented to enhance the bioactivities of the roe protein hydrolysates.

Therefore, the subject of this study is to examine the possible antiproliferative function of fish roe hydrolysates of giant grouper (*Epinephelus lanceolatus*) against oral cancer cells and explore its detailed mechanisms in terms of cell viability, cell cycle analysis, apoptosis, reactive oxygen species (ROS)/superoxide generations, and mitochondrial membrane potential.

## 2. Materials and Methods

### 2.1. Preparation of Defatted Roe

Fresh fish roes of giant groupers (*E. lanceolatus*) were obtained from farm ponds in Pingtung, Taiwan, during July 2013. The samples were placed in ice and transported to the Department of Seafood Science, Kaohsiung Marine University, within 1 h. The whole roes were cleaned using cool water (4°C) and homogenized in a cool room. The homogenized roes were then freeze-dried. Afterward, lipids of the dried roe powders were extracted as described previously [26]. In brief, each 100 g freeze-dried homogenized roes were added to 300 mL hexane for 2 h fat extraction. This procedure was repeated three times. Moreover, the solvents were evaporated by vacuum concentration. The defatted roe protein samples were then freeze-dried. The defatted roe powders were kept in sealed polyethylene bag and then placed at −40°C until use.

### 2.2. Preparation of Roe Protein Hydrolysate

Roe protein hydrolysate (RPH) was prepared from defatted grouper roe powder using Protease N (Amano Pharmaceutical Co., Nagoya, Japan). The defatted sample (5 g dry matter) was suspended in a 250 mL of pH 8 phosphate buffer. The hydrolysis reaction was initiated by the ratio of Protease N/roe sample at 1 : 100 (w/w solid matter). The reaction was conducted at pH 8 and 50°C for 9 h. The enzymatic hydrolysis was ended by heating the mixtures at 90°C for 10 min to inactivate the protease activity. The solution containing hydrolysate was centrifuged at 5000 g for 10 min at 4°C (05PR-22 centrifuge, Hitachi, Tokyo, Japan). Then, the supernatants were desalted and lyophilized to dried RPH for storage at −40°C.

### 2.3. Preparation of Ultrafiltrates from RPH through Centrifugal Ultrafiltration Filters

The lyophilized RPH (obtained from 9 h Protease N hydrolysis) was subsequently dissolved in deionized distilled water. The solution containing RHP was processed through centrifugal ultrafiltration (UF) filters (Millipore, Bedford, MA, USA) as described previously [5]. RPH solution (12 mL) was first passed through a centrifugal filter with 10 kDa MWCO and then its permeate was passed through the UF membranes with 5 kDa MWCO. The permeates (ultrafiltrated roe hydrolysates, URH) with molecular size below 5 kDa were obtained. The URH filtrate was lyophilized and stored at −40°C until use.

### 2.4. Amino Acid Analysis of URH

The URH were hydrolyzed with 6 N HCl at 110°C for 24 h under vacuum. The amino acid analysis was performed using the Pico-Tag system (Waters, Milford, MA) as described [27].

### 2.5. Cell Cultures

Two human oral cancer cell lines (Ca9-22 and CAL 27) were available from the Japanese Collection of Research Bioresources (JCRB) Cell Bank (National Institute of Biomedical Innovation, Osaka, Japan) and the American Type Culture Collection (ATCC; Virginia, USA), respectively [28]. Cells were maintained in DMEM/F12 (3 : 2) medium (Gibco, Grand Island, NY, USA) supplemented with 10% fetal bovine serum (Gibco), 100 U/mL penicillin, 100 *μ*g/mL streptomycin, and 0.03% glutamine under the humidified incubator at 37°C with 5% CO_2_.

### 2.6. Cell Viability

URH was dissolved in culture medium for cell treatment. Cells were plated at 4000 cells/well in 96-well plates. After plating overnight, cells were treated with URH at indicated concentrations (0, 0.25, 0.5, 0.75, 1, 1.5, 2, and 2.5 mg/mL) for 24 h. Then, cellular ATP level was analyzed by the ATP-lite Luminescence ATP Detection Assay System (PerkinElmer Life Sciences, Boston, MA, USA) according to the manufacturer's instructions [29]. Finally, the luminescence was detected using a microplate luminometer (CentroPRO LB 962; Berthold, ND, USA).

### 2.7. Cell Cycle Distribution

Cellular DNA was detected by propidium iodide (PI) (Sigma, St. Louis, MO, USA) [30]. In brief, 3 × 10^5^ cells per well in 6-well plates were plated overnight. Cells were treated with 0, 0.5, 1, 1.5, 2, and 2.5 mg/mL of URH for 24 h. After harvest, cells were washed with PBS and fixed overnight with 70% ethanol. Finally, the cells were resuspended in 50 *μ*g/mL PI in PBS for 30 min at 37°C in darkness. Cell cycle distribution was determined by a flow cytometer (BD Accuri*™* C6; Becton-Dickinson, Mansfield, MA, USA) and a BD Accuri C6 Software (version 1.0.264).

### 2.8. Apoptosis by Annexin V/PI

The apoptosis-like (sub-G1) status was further examined by annexin V (Strong Biotect Corporation, Taipei, Taiwan)/PI as described [31]. In brief, 3 × 10^5^ cells per well in 6-well plates were plated for 24 h. Cells were treated with the indicated concentrations of URH for 24 h. After drug treatment, cells were incubated with 100 *μ*L binding buffer containing 2 *μ*L of annexin-V-fluorescein isothiocyanate (FITC) stock (0.25 *μ*g/*μ*L) and 2 *μ*L of PI stock (1 mg/mL) for 30 min. Finally, it was suspended with 400 *μ*L PBS for analysis by a flow cytometer (BD Accuri C6) and its software.

### 2.9. Intracellular ROS

The fluorescent dye 2′,7′-dichlorodihydrofluorescein diacetate (DCFH-DA) was used to detect ROS [19]. 3 × 10^5^ cells per well in 6-well plates in 2 mL medium were plated for 24 h. Cells were treated with different concentrations of URH for 6 h. After PBS washing, 100 nM DCFH-DA in PBS was added to cells in 6-well plates under an incubator for 30 min. After harvest, PBS washing, and centrifugation, cells were resuspended in 1 mL PBS for analysis by a flow cytometer (BD Accuri C6) and its software.

### 2.10. Intracellular Superoxide

MitoSOX*™* Red mitochondrial superoxide indicator (Molecular Probes, Invitrogen, Eugene, OR, USA) was reported to be the fluorescent dye for mitochondrial superoxide [32]. Assessing mitochondrial redox status has been detected by flow cytometric methods [33]. With a slight modification, 3 × 10^5^ cells per well in 6-well plates in 2 mL medium were plated for 24 h. Cells were treated with different concentrations of URH for 1 h. Subsequently, 5 *μ*M MitoSOX was added to cells in 6-well plates under an incubator for 10 min. After harvest, PBS washing, and centrifugation, cells were resuspended in 1 mL PBS for analysis by a flow cytometer (BD Accuri C6) and its software.

### 2.11. Mitochondrial Membrane Potential (MMP)

MitoProbe*™* DiOC_2_(3) assay kit (Invitrogen, Eugene, OR, USA) was used to measure MMP as described previously [34]. Briefly, 3 × 10^5^ cells in 2 mL medium per well in 6-well plate were plated and incubated for 24 h. Cells were treated with URH treatment for 24 h. Subsequently, 50 nM DiOC_2_(3) was added per well under an incubator for 30 min. After harvest, cells were resuspended in 1 mL PBS for analysis by a flow cytometer (BD Accuri C6) and its software.

### 2.12. Statistical Analysis

The significance of differences was evaluated by Student's* t*-test in SigmaPlot 10 software (Systat Software Inc., San Jose, CA, USA). All data were compared with controls. *∗* and *∗∗*, respectively, indicate *p* < 0.05 and *p* < 0.001 against control.

## 3. Results

### 3.1. Amino Acid Composition of URH

As shown in Table 1, the amino acid composition of URH indicates that URH was composed of full kind of amino acids after purification processes.

### 3.2. Antiproliferation of URH

**Figure 1:**
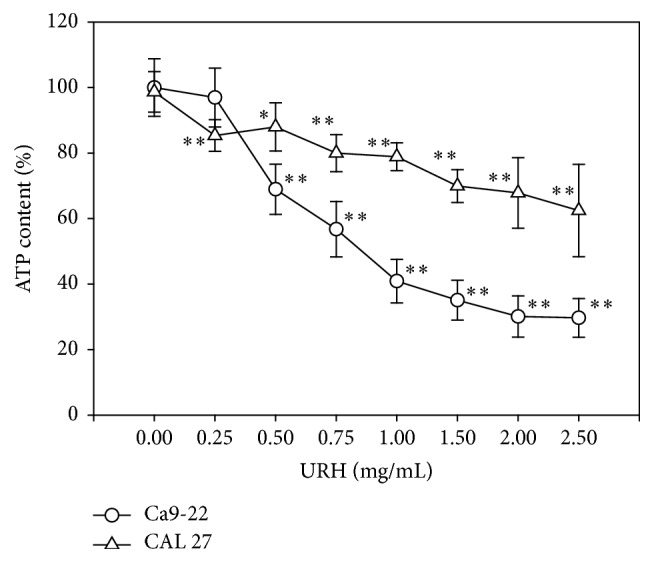
Cell viabilities of two URH-treated oral cancer cells. Oral cancer (Ca9-22 and CAL 27) cells were treated with 0, 0.5, 0.75, 1, 1.5, 2, and 2.5 mg/mL of URH for 24 h incubation. The cell viability was measured by the ATP assay. Data, means ± SDs (*n* = 6). ^*∗*^ *p* < 0.05; ^*∗∗*^ *p* < 0.001 against control.

With the cell viability (%) in terms of ATP content measurement (Figure 1), two oral cancer cells (Ca9-22 and CAL 27) at indicated concentrations of URH were dose-responsively decreased (*p* < 0.05–0.001 compared to the control). The IC_50_ values of URH at 24 h treatment for oral cancer Ca9-22 cells were 0.85 mg/mL and IC_50_ value was undetectable for CAL 27 cells.

### 3.3. Morphology Change of URH

**Figure 2:**
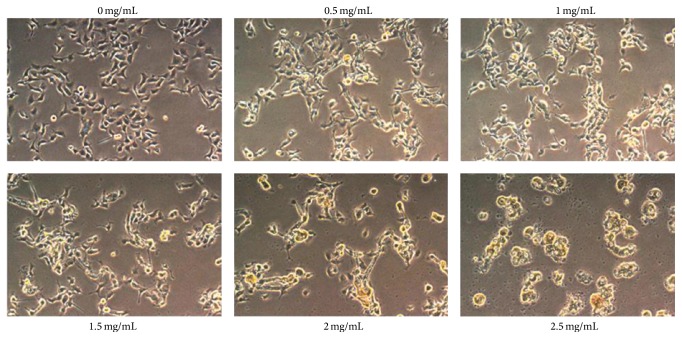
Cell morphology of URH-treated oral cancer Ca9-22 cells. Cells were treated with 0, 0.5, 0.75, 1, 1.5, 2, and 2.5 mg/mL of URH for 24 h incubation. Cell images were captured at 100x magnification.

The cell morphology of URH-treated oral cancer Ca9-22 cells was shown in Figure 2. The morphological features of apoptosis, including apoptotic bodies and shrinkage of the cells, appeared at higher concentration of URH.

### 3.4. Cell Cycle Disturbance of URH

**Figure 3:**
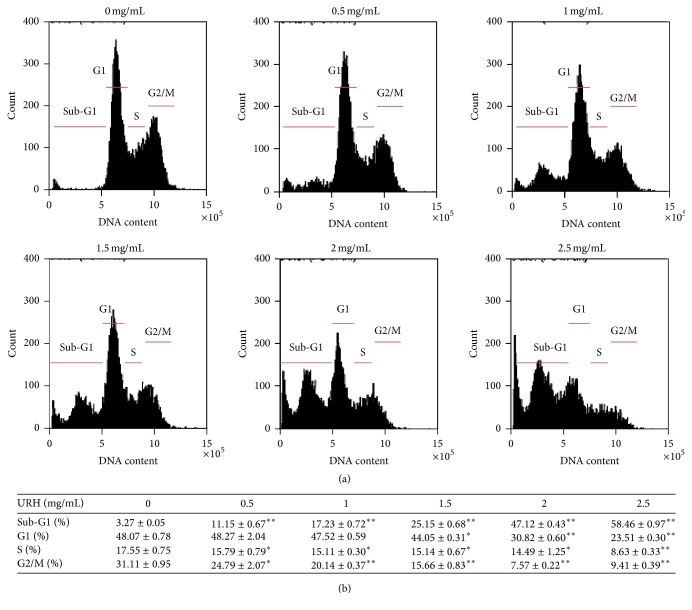
The cell cycle changes of URH-treated oral cancer Ca9-22 cells. Cells were treated with 0, 0.5, 1, 1.5, 2, and 2.5 mg/mL of URH for 24 h. (a) Representative cell cycle distribution patterns of flow cytometry of URH-treated Ca9-22 cells. The cell cycle phases were labeled in each panel. (b) Quantification analysis for the cell cycle phases in Figure 3(a). Data, mean ± SD (*n* = 3). ^*∗*^ *p* < 0.05 and ^*∗∗*^ *p* < 0.001 against control.

As shown in Figure 3(a), the flow cytometry-based cell cycle distribution patterns of URH-treated oral cancer Ca9-22 cells were displayed. After URH treatment (Figure 3(b)), the sub-G1 (%) of URH-treated (0–2.5 mg/mL) Ca9-22 cells were dose-responsively increased (*p* < 0.001). In contrast, the G0/G1, S, and G2/M (%) of URH-treated Ca9-22 cells were dose-responsively decreased (*p* < 0.05–0.001).

### 3.5. Annexin V/PI-Based Apoptosis of URH

**Figure 4:**
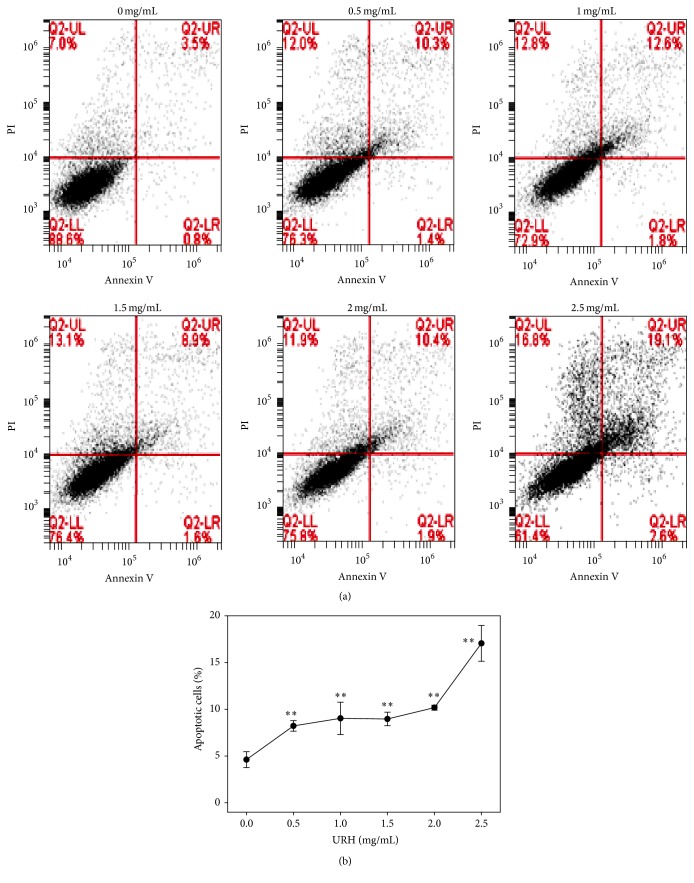
Annexin V/PI-based apoptosis of URH-treated oral cancer Ca9-22 cells. Ca9-22 cells were treated with 0, 0.5, 1, 1.5, 2, and 2.5 mg/mL of URH for 24 h. (a) Representative results of flow cytometry-based annexin V/PI double staining of URH-treated Ca9-22 cells. Annexin V (+)/PI (+) and annexin V (+)/PI (−) were calculated as the apoptosis (+) in each panel. (b) Quantification analysis of apoptosis for URH-treated Ca9-22 cells in Figure 4(a). Data, mean ± SD (*n* = 3). ^*∗∗*^ *p* < 0.001 against control.

To further examine the role of apoptosis, the flow cytometry-based annexin V/PI patterns of URH-treated oral cancer Ca9-22 cells were performed (Figure 4(a)). As shown in Figure 4(b), the annexin V-positive intensities (%) for URH-treated (0–2.5 mg/mL) Ca9-22 cells were dose-responsively increased (*p* < 0.05–0.001).

### 3.6. ROS Generation of URH

**Figure 5:**
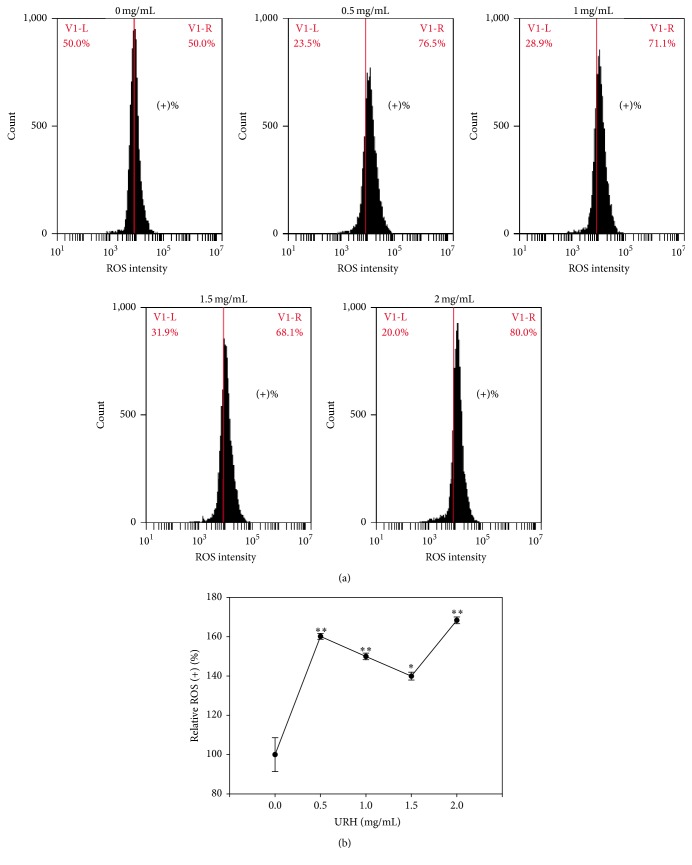
ROS generation of URH-treated oral cancer Ca9-22 cells. Cells were treated with 0, 0.5, 1, 1.5, and 2 mg/mL of URH for 6 h. (a) Representative ROS patterns of flow cytometry for URH-treated Ca9-22 cells. In each panel, the right side labeled with (+)% indicates the ROS-positive region. (b) Quantification analysis of relative ROS intensity in Figure 5(a). Data, mean ± SD (*n* = 3). ^*∗*^ *p* < 0.05 and ^*∗∗*^ *p* < 0.001 against control.

Since some apoptosis-inducible drugs were associated with ROS generation [35–38], the role of oxidative stress in URH-treated Ca9-22 cells was examined in terms of ROS detection. As shown in Figure 5(a), the flow cytometry-based ROS staining patterns of URH-treated Ca9-22 cells at 6 h incubation were displayed. As shown in Figure 5(b), the relative ROS-positive intensities (%) of URH-treated (0–2 mg/mL) Ca9-22 cells were dose-responsively induced (*p* < 0.05–0.001).

### 3.7. Superoxide Generation of URH

**Figure 6:**
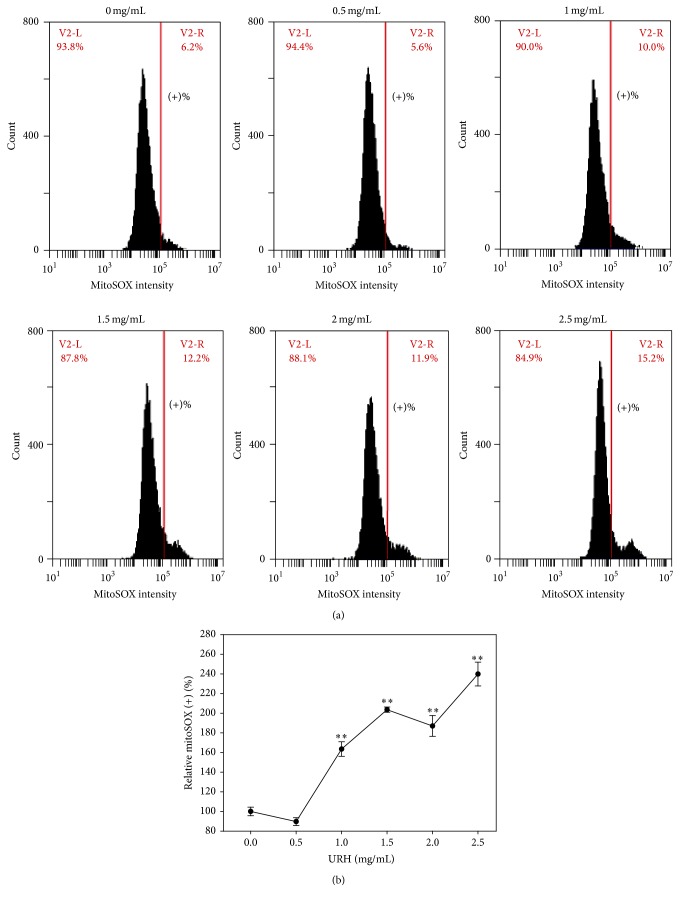
Superoxide generation of URH-treated oral cancer Ca9-22 cells. (a) Ca9-22 cells treated with 0, 0.5, 1, 1.5, 2, and 2.5 mg/mL of URH for 1 h were stained with MitoSOX dye. The right side labeled with (+)% indicates the MitoSOX-positive region in each panel. (b) Quantification analysis of relative MitoSOX (+) fluorescent intensity (%). Data, mean ± SD (*n* = 3). ^*∗∗*^ *p* < 0.001 against control.

The role of oxidative stress in URH-treated Ca9-22 cells was examined in terms of superoxide detection. As shown in Figure 6(a), the flow cytometry-based superoxide staining (MitoSOX) patterns of URH-treated (0–2.5 mg/mL) Ca9-22 cells at 1 h incubation were displayed. As shown in Figure 6(b), the relative MitoSOX-positive intensities (%) of URH-treated (0–2.5 mg/mL) Ca9-22 cells were dose-responsively induced (*p* < 0.05–0.001).

### 3.8. MMP of URH

**Figure 7:**
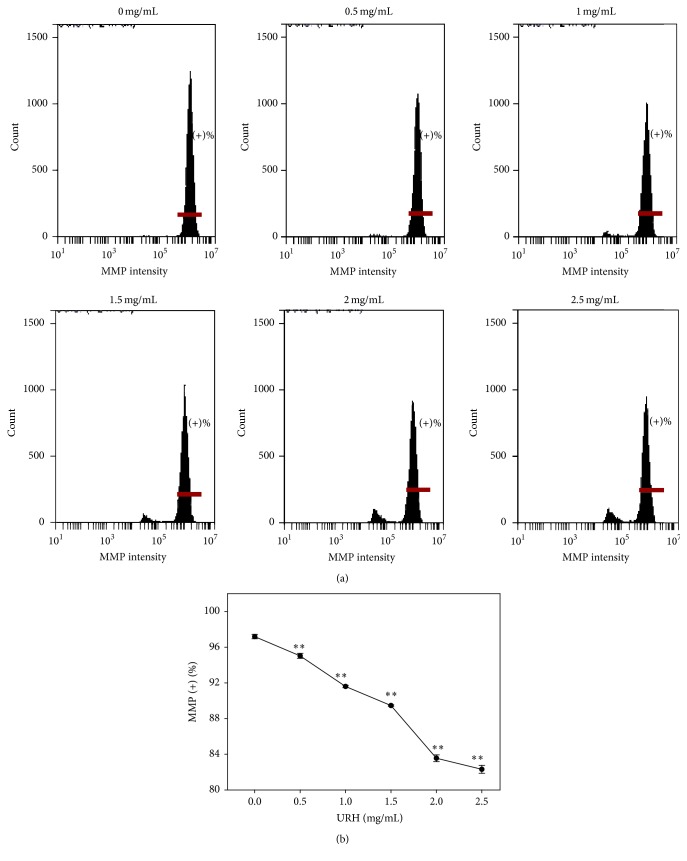
MMP change of URH-treated oral cancer Ca9-22 cells. Cells were treated with 0, 0.5, 1, 1.5, 2, and 2.5 mg/mL of URH for 24 h. (a) Representative MMP patterns of flow cytometry for URH-treated Ca9-22 cells. In each panel, the horizontal line labeled with (+)% in the right side indicates the MMP-positive region. (b) Quantification analysis of MMP intensity ((+)%) in Figure 7(a). Data, mean ± SD (*n* = 3). ^*∗∗*^ *p* < 0.001 against control.

The role of oxidative stress in URH-treated Ca9-22 cells was also examined in terms of MMP by flow cytometry. As shown in Figure 7(a), the MMP staining patterns of URH-treated Ca9-22 cells at 24 h incubation were displayed. As shown in Figure 7(b), the MMP-positive intensities (%) of URH-treated (0–2.5 mg/mL) Ca9-22 cells were dose-responsively decreased (*p* < 0.001) (Figure 7(b)).

## 4. Discussion

Fish protein hydrolysates were well-known for the antioxidant property. Although most studies of fish protein hydrolysates had been reported, the possible anticancer effect was less addressed. In current study, we chose the* Epinephelus lanceolatus*-derived roe hydrolysates (URH) and validated their antiproliferative effect against oral cancer cells. We found that URH induced antiproliferation, sub-G1 accumulation, apoptosis, ROS generation, and mitochondrial depolarization of oral cancer cells.

In the current study, the fish roe protein hydrolysates-derived URH is the ultrafiltration fraction with low molecular weights (MW) (<5 kDa) and it showed that the IC_50_ values at 24 h treatment for oral cancer Ca9-22 cells were 0.85 mg/mL in terms of ATP assay. The IC_30_ (70% viability) values of URH at 24 h treatment for oral cancer Ca9-22 and CAL 27 cells were 0.5 and 1.5 mg/mL, respectively. Similarly, the muscle tissue-derived loach (*Misgurnus anguillicaudatus*) protein hydrolysates (LPH) prepared by papain digestion displayed differential antiproliferative activities against breast, colon, and liver cancer cells for different ultrafiltration fractions with different MW ranges [21]. Based on MTS assay, the IC_50_ values of LPH-III (MW ranging from 3 to 5 kDa) at 96 h treatment were 33, 15, and 22 mg/mL for breast (MCF-7), colon (Caco-2), and liver (HepG2) cancer cells, respectively. IC_50_ values of LPH-IV (MW ranging from <3 kDa) at 96 h treatment were 16, 10, and 13 mg/mL for MCF-7, Caco-2, and HepG2 cancer cells, respectively. Accordingly, the LPH fractions with the low MW ranging from <5 kDa (LPH-III and LPH-IV) have detectable IC_50_ values at 96 h treatment. In contrast, the IC_50_ values of the LPH fractions with the high MW (5–10 and >10 kDa) were undetectable. Therefore, antiproliferative activities against cancer cells may be varied with different ultrafiltration fractions with different MW ranges. Low MW protein hydrolysates seem to be more potential to anticancer cell proliferation.

The antiproliferative effect of several hydrolysates from different parts of the marine species had been reported in other cancer types such as breast [20], colon, and liver [39] cancers. For example, peptide-rich fish hydrolysates, derived from fresh filleting by-products or headed and gutted by-catches of blue whiting, exerted a significant antiproliferative activity at 1 mg/mL for 72 h treatment with growth inhibition of 22.3–26.3% on breast cancer MCF-7/6 cells and 13.5–29.8% on breast cancer MDA-MB-231 cells using MTS assay [20]. Because these protein hydrolysate studies mentioned above were derived from different parts and species of fishes and they were treated with different incubation times, it was suitable to compare their drug sensitivity between each other. In general, our experiment was performed in shorter treatment time and displayed the lower IC_50_ values of fish protein hydrolysates (URH) in inhibiting proliferation of oral cancer cells. It is also possible that drug sensitivity of fish protein hydrolysates in anticancer cell effect may be cancer cell-type dependent. Moreover, it was warranted for further investigation that the fish protein hydrolysates from roe and other parts may have different potentials for antiproliferation of oral cancer cells.

Protein hydrolysates from many species were reported to have the antioxidant property [5–13]. The dual roles of antioxidants can explain why protein hydrolysates with antioxidant property also display anticancer effect [40]. Oxidative stress had been reviewed to regulate the endoplasmic reticulum stress [41], autophagy [42], and apoptosis [43], leading to cell death. For exogenous antioxidants, it may behave like the double-edged swords in cellular redox state; that is, it is protective at physiologic doses but it is harmful at high doses [40]. In current study, we provided evidence for the apoptosis effect of URH in oral cancer Ca9-22 cells, such as sub-G1 accumulation and annexin V/PI staining (Figures 3 and 4), which were coupled with high ROS, superoxide generations, and mitochondrial depolarization; that is, the correlation values (*R* ^2^) are 0.8435, 0.6294, and 0.6782 for ROS (+), mitoSOX (+), and MMP (−) versus apoptosis (annexin V (+)), respectively, although the effect of lower doses of URH was not examined in our study. Similarly, the grape seed extract (GSE) displayed the normal proliferation at low doses but demonstrated the antiproliferation for oral cancer cells at high doses [16]. Moreover, GSE at high doses displayed high ROS generation and mitochondrial depolarization than those of the low doses [16]. Therefore, the differential oxidative stress may partly contribute the dual roles of antioxidants.

## 5. Conclusion

URH is the ultrafiltration fraction of fish roe protein hydrolysates with low MW. In current study, we firstly demonstrated that URH can inhibit cell proliferation of two oral cancer cells (Ca9-22 and CAL 27). URH also induced the characters of apoptosis of oral cancer cells such as apoptotic morphology change, sub-G1 accumulation, and annexin V/PI positive expression. This antiproliferative mechanism includes the ROS and superoxide generations and mitochondrial depolarization. Therefore, these results suggest that URH has an apoptosis-based anticancer potential for oral cancer therapy.

## Figures and Tables

**Table 1:** Amino acid composition^*∗*^ of URH.

Amino acid	(%)
Asp + Asn	9.79
Glu + Gln	12.76
Ser	7.11
Gly	7.84
His	1.71
Arg	3.45
Thr	5.55
Ala	11.29
Pro	8.21
Tyr	2.49
Val	6.57
Met	1.67
Cys	0.31
Ile	4.79
Leu	7.58
Phe	2.66
Lys	6.21

^*∗*^Data are the mean values of duplicate determinations expressed as milligram of amino acid per 100 mg of URH.

## References

[1] Warnakulasuriya S. (2009). Global epidemiology of oral and oropharyngeal cancer. *Oral Oncology*.

[2] Petersen P. E. (2009). Oral cancer prevention and control—the approach of the World Health Organization. *Oral Oncology*.

[3] Yen C.-Y., Huang C.-Y., Hou M.-F. (2013). Evaluating the performance of fibronectin 1 (FN1), integrin *α*4*β*1 (ITGA4), syndecan-2 (SDC2), and glycoprotein CD44 as the potential biomarkers of oral squamous cell carcinoma (OSCC). *Biomarkers*.

[4] Tanaka T., Tanaka M., Tanaka T. (2011). Oral carcinogenesis and oral cancer chemoprevention: a review. *Pathology Research International*.

[5] Yang J.-I., Ho H.-Y., Chu Y.-J., Chow C.-J. (2008). Characteristic and antioxidant activity of retorted gelatin hydrolysates from cobia (*Rachycentron canadum*) skin. *Food Chemistry*.

[6] Yang J.-I., Liang W.-S., Chow C.-J., Siebert K. J. (2009). Process for the production of tilapia retorted skin gelatin hydrolysates with optimized antioxidative properties. *Process Biochemistry*.

[7] Chow C.-J., Yang J.-I. (2008). The effect of process variables for production of cobia (*Rachycentron canadum*) skin gelatin hydrolysates with antioxidant properties. *Journal of Food Biochemistry*.

[8] Li X., Luo Y., Shen H., You J. (2012). Antioxidant activities and functional properties of grass carp (*Ctenopharyngodon idellus*) protein hydrolysates. *Journal of the Science of Food and Agriculture*.

[9] Elavarasan K., Naveen Kumar V., Shamasundar B. A. (2014). Antioxidant and functional properties of fish protein hydrolysates from fresh water carp (*Catla catla*) as influenced by the nature of enzyme. *Journal of Food Processing and Preservation*.

[10] Chalamaiah M., Dinesh Kumar B., Hemalatha R., Jyothirmayi T. (2012). Fish protein hydrolysates: proximate composition, amino acid composition, antioxidant activities and applications: a review. *Food Chemistry*.

[11] Balaswamy K., Prabhakara Rao P. G., Narsing Rao G., Jyothirmayi T. (2011). Functional properties of roe protein hydrolysates from *Catla catla*. *Electronic Journal of Environmental, Agricultural, and Food Chemistry*.

[12] Galla N. R., Pamidighantam P. R., Akula S., Karakala B. (2012). Functional properties and in vitro antioxidant activity of roe protein hydrolysates of *Channa striatus* and *Labeo rohita*. *Food Chemistry*.

[13] Rao G. N. (2014). Physico-chemical, functional and antioxidant properties of roe protein concentrates from *Cyprinus carpio* and *Epinephelus tauvina*. *Journal of Food and Pharmaceutical Sciences*.

[14] Lee J.-C., Hou M.-F., Huang H.-W. (2013). Marine algal natural products with anti-oxidative, anti-inflammatory, and anti-cancer properties. *Cancer Cell International*.

[15] Najafian L., Babji A. S. (2012). A review of fish-derived antioxidant and antimicrobial peptides: their production, assessment, and applications. *Peptides*.

[16] Yen C.-Y., Hou M.-F., Yang Z.-W. (2015). Concentration effects of grape seed extracts in anti-oral cancer cells involving differential apoptosis, oxidative stress, and DNA damage. *BMC Complementary and Alternative Medicine*.

[17] Yeh C.-C., Tseng C.-N., Yang J.-I. (2012). Antiproliferation and induction of apoptosis in Ca9-22 oral cancer cells by ethanolic extract of *Gracilaria tenuistipitata*. *Molecules*.

[18] Yen Y.-H., Farooqi A. A., Li K.-T. (2014). Methanolic extracts of *Solieria robusta* inhibits proliferation of oral cancer Ca9-22 cells via apoptosis and oxidative stress. *Molecules*.

[19] Yeh C.-C., Yang J.-I., Lee J.-C. (2012). Anti-proliferative effect of methanolic extract of *Gracilaria tenuistipitata* on oral cancer cells involves apoptosis, DNA damage, and oxidative stress. *BMC Complementary and Alternative Medicine*.

[20] Picot L., Bordenave S., Didelot S. (2006). Antiproliferative activity of fish protein hydrolysates on human breast cancer cell lines. *Process Biochemistry*.

[21] You L., Zhao M., Liu R. H., Regenstein J. M. (2011). Antioxidant and antiproliferative activities of loach (*Misgurnus anguillicaudatus*) peptides prepared by papain digestion. *Journal of Agricultural and Food Chemistry*.

[22] Chi C.-F., Hu F.-Y., Wang B., Li T., Ding G.-F. (2015). Antioxidant and anticancer peptides from the protein hydrolysate of blood clam (*Tegillarca granosa*) muscle. *Journal of Functional Foods*.

[23] Umayaparvathi S., Meenakshi S., Vimalraj V., Arumugam M., Sivagami G., Balasubramanian T. (2014). Antioxidant activity and anticancer effect of bioactive peptide from enzymatic hydrolysate of oyster (*Saccostrea cucullata*). *Biomedicine & Preventive Nutrition*.

[24] Xue Z. H., Wen H. C., Zhai L. J. Y. (2015). Antioxidant activity and anti-proliferative effect of a bioactive peptide from chickpea (*Cicer arietinum* L.). *Food Research International*.

[25] Hseu J.-R., Hwang P.-P., Ting Y.-Y. (2004). Morphometric model and laboratory analysis of intracohort cannibalism in giant grouper *Epinephelus lanceolatus* fry. *Fisheries Science*.

[26] Bligh E. G., Dyer W. J. (1959). A rapid method of total lipid extraction and purification. *Canadian Journal of Biochemistry and Physiology*.

[27] Sato K., Tsukamasa Y., Imai C., Ohtsuki K., Shimizu Y., Kawabata M. (1992). Improved method for identification and determination of *ε*-(*γ*-glutamyl)-lysine cross-link in protein using proteolytic digestion and derivatization with phenyl isothiocyanate followed by high performance liquid chromatography separation. *Journal of Agricultural and Food Chemistry*.

[28] Jiang L., Ji N., Zhou Y. (2009). CAL 27 is an oral adenosquamous carcinoma cell line. *Oral Oncology*.

[29] Wei J., Stebbins J. L., Kitada S. (2011). An optically pure apogossypolone derivative as potent pan-active inhibitor of anti-apoptotic Bcl-2 family proteins. *Frontiers in Oncology*.

[30] Chen B.-H., Chang H.-W., Huang H.-M. (2011). (-)-Anonaine induces DNA damage and inhibits growth and migration of human lung carcinoma H1299 cells. *Journal of Agricultural and Food Chemistry*.

[31] Chiu C.-C., Liu P.-L., Huang K.-J. (2011). Goniothalamin inhibits growth of human lung cancer cells through DNA damage, apoptosis, and reduced migration ability. *Journal of Agricultural and Food Chemistry*.

[32] Mukhopadhyay P., Rajesh M., Yoshihiro K., Haskó G., Pacher P. (2007). Simple quantitative detection of mitochondrial superoxide production in live cells. *Biochemical and Biophysical Research Communications*.

[33] Li R., Jen N., Yu F., Hsiai T. K. (2011). Assessing mitochondrial redox status by flow cytometric methods: vascular response to fluid shear stress. *Current Protocols in Cytometry*.

[34] Yen C.-Y., Chiu C.-C., Haung R.-W. (2012). Antiproliferative effects of goniothalamin on Ca9-22 oral cancer cells through apoptosis, DNA damage and ROS induction. *Mutation Research/Genetic Toxicology and Environmental Mutagenesis*.

[35] Yen C.-Y., Lin M.-H., Liu S.-Y. (2011). Arecoline-mediated inhibition of AMP-activated protein kinase through reactive oxygen species is required for apoptosis induction. *Oral Oncology*.

[36] Han M. H., Park C., Jin C.-Y. (2013). Apoptosis induction of human bladder cancer cells by sanguinarine through reactive oxygen species-mediated up-regulation of early growth response gene-1. *PLoS ONE*.

[37] Raj L., Ide T., Gurkar A. U. (2011). Selective killing of cancer cells by a small molecule targeting the stress response to ROS. *Nature*.

[38] Ding H., Han C., Guo D. (2009). Selective induction of apoptosis of human oral cancer cell lines by avocado extracts via a ROS-mediated mechanism. *Nutrition and Cancer*.

[39] Kannan A., Hettiarachchy N. S., Marshall M., Raghavan S., Kristinsson H. (2011). Shrimp shell peptide hydrolysates inhibit human cancer cell proliferation. *Journal of the Science of Food and Agriculture*.

[40] Bouayed J., Bohn T. (2010). Exogenous antioxidants—double-edged swords in cellular redox state: health beneficial effects at physiologic doses versus deleterious effects at high doses. *Oxidative Medicine and Cellular Longevity*.

[41] Farooqi A. A., Li K.-T., Fayyaz S. (2015). Anticancer drugs for the modulation of endoplasmic reticulum stress and oxidative stress. *Tumor Biology*.

[42] Farooqi A. A., Fayyaz S., Hou M.-F., Li K.-T., Tang J.-Y., Chang H.-W. (2014). Reactive oxygen species and autophagy modulation in non-marine drugs and marine drugs. *Marine Drugs*.

[43] Matés J. M., Segura J. A., Alonso F. J., Márquez J. (2012). Oxidative stress in apoptosis and cancer: an update. *Archives of Toxicology*.

